# “Everything Will Be All Right!” National and European Identification as Predictors of Positive Expectations for the Future During the COVID-19 Emergency

**DOI:** 10.3389/fpsyg.2021.723518

**Published:** 2021-10-21

**Authors:** Silvia Moscatelli, Anna Rita Graziani, Lucia Botindari, Stefano Ciaffoni, Michela Menegatti

**Affiliations:** ^1^Department of Psychology, Alma Mater Studiorum University of Bologna, Bologna, Italy; ^2^Department of Communication and Economics, University of Modena and Reggio Emilia, Reggio Emilia, Italy; ^3^SAIS Bologna Center, John Hopkins University, Bologna, Italy

**Keywords:** COVID-19, national identification, European identification, expectations for the future, trust in institutions

## Abstract

During the first national lockdown imposed in a Western country to reduce the impact of the COVID-19 pandemic, many Italians tried to boost their spirits by hanging hand-drawn rainbows with the slogan “Everything will be all right” from their windows. To understand which processes might have nurtured their positive views about the future during the pandemic, the present study (*N*=846), building upon social identity research, examined the relationships among Italians’ identification with their country and with the superordinate entity of the European Union (EU), trust in the main institutions in charge of managing the crisis (i.e., the Italian government, the EU, and the scientific community), and beliefs that the COVID-19 crisis would eventually result in the improvement of society. Structural equation modeling analyses showed that identification with Italians and Europeans had positive direct associations with positive expectations about humankind. Identification with Europeans was also directly related to positive expectations about Italian leaders and the strengthening of the EU through the crisis. Trust in the Italian government and, to a lower extent, trust in the EU mediated some of these associations. These findings suggest that governments should actively promote national and European identification to help citizens counter the negative psychological impact of the pandemic and maintain positive views of the future.

## Introduction

Since the end of 2019, COVID-19, a new acute respiratory syndrome in humans, has affected the lives of many people in different ways all over the world (Phelan et al., 2020; WHO, 2020). Italy was the first Western country to be hit by the COVID-19 outbreak and, at the beginning of March 2020, presented the world’s highest intensity of coronavirus infections (Taddei, 2020). To flatten the infection curve, the Italian Prime Minister imposed a strict nationwide lockdown on March 9, 2020: The operations of schools, universities, and all non-essential industries and businesses were halted, and free movement was forbidden, except for essential reasons. At the time, the eyes of the entire Western world were on Italy, and media around the world shared the images of the country’s desolate cities. However, along with the videos of empty streets and cafés, a message of hope went viral: Many children and adults stuck at home began to hang hand-drawn rainbows accompanied by the slogan of reassurance “Andrà tutto bene” (Everything will be all right) from their windows and balconies. Italians were attempting to boost their spirits and communicate their hopes that the COVID-19 outbreak would be over soon and might even have positive outcomes.

Which psychosocial mechanisms might have contributed to nourishing the hope that the future could be better after all? To date, most studies have highlighted the possible negative impacts of isolation and lockdown on individual well-being and mental health (Brooks et al., 2020; Cao et al., 2020; Hossain et al., 2020; Kachanoff et al., 2020) or focused on the possible antecedents of people’s compliance with pandemic restrictions (Lalot et al., 2020; Tunçgenç et al., 2021). In contrast, the present research aims to understand what processes might nurture individuals’ positive expectations about the future during the COVID-19 pandemic.

Optimism, which represents a general tendency to expect favorable outcomes (Scheier and Carver, 1985), has been found to protect against COVID-19-related stress (Genç and Arslan, 2021; Yıldırım and Arslan, 2021). Therefore, identifying the factors that support positive expectations for life after the coronavirus crisis has important implications for helping people cope with difficulties related to the pandemic. Whereas optimism is generally conceived as a personality trait and thus a rather stable individual characteristic (Scheier and Carver, 1985), in this research, we focused on possible antecedents of more specific beliefs that the COVID-19 crisis might eventually result in the improvement of society and humankind.

To this end, we built upon research conducted in the social identity framework, which highlighted that the subjective sense of belonging to meaningful groups affects how well people cope with crises (Jetten et al., 2020), leads them to act selflessly for the greater good (Drury, 2018; Haslam et al., 2018), and fuels mutual trust with the group and its authorities (e.g., Haslam, 2001; Giessner and van Knippenberg, 2008; Cruwys et al., 2020). Accordingly, this study examined the relationships among Italians’ identifications with meaningful social groups [i.e., their country and the superordinate entity of the European Union (EU)], trust in some of the main institutions responsible for managing the crisis – the Italian government, the European Union, and the scientific community – and positive expectations for the future during the first national lockdown.

### Dealing With the COVID-19 Crisis Through Social Identification

Italian Prime Minister Giuseppe Conte, concluding the dramatic speech that announced the first general lockdown, said: “We are all part of the same community […]. We keep distant today to hug tighter tomorrow. We will run together again; we will make it together.” In social-psychological terms, he used Italians’ shared identity as leverage to obtain greater compliance with the new restrictions as well as to preserve optimism and hope for the future. This communicative strategy, characterized by the emphasis on a sense of shared group membership – that is, a sense of shared social identity, or “we-ness” – in citizens, has been successfully employed during the pandemic by political and religious leaders such as Pope Francis (Scardigno et al., 2021), or Jacinda Ardern, New Zealand’s Prime Minister (Haslam et al., 2021).

Indeed, relying on the group rather than the individual can be an effective way to fight the coronavirus or minimize its negative consequences, whether health, social, or economic (Jetten et al., 2020). According to the social identity approach (Tajfel and Turner, 1986; Turner et al., 1994), a person can self-define as an individual – in terms of “I” (personal identity) – as well as a member of a group – part of a “we.” When people think about themselves as part of a specific group, their attitudes and behaviors are strongly affected by their social identities (Haslam, 2001). Several studies demonstrated that social identification, along with a more general sense of connectedness, improves physical and mental health (e.g., Wakefield et al., 2017; Haslam et al., 2018). Belonging to meaningful groups fulfills individuals’ needs for control, self-esteem, sense of belonging, and meaning (Hogg and Abrams, 1990, 1993; Baumeister and Leary, 1995). Social identification is related to decreased depression and stress and helps people cope with difficulties on both a psychological and physical level (Sani et al., 2012; Wakefield et al., 2017). Moreover, feeling that one is part of a group and knowing that one can rely on fellow group members fosters empathy and solidarity, leading to selfless behavior and reciprocal helping (Drury, 2018). From the perspective of terror management theory (Greenberg et al., 1997), one might also argue that the increased mortality salience due to the COVID-19 pandemic might enhance ingroup identification as a way for individuals to reduce anxiety and seek transcendence through the stronger sense of belonging to something greater and longer-lasting than the individual self (Castano and Dechesne, 2005; Pyszczynski et al., 2021).

More in general, during national crises, facing a collective threat and perceiving that the group shares a common fate can nurture a greater sense of “we-ness” and lead people to focus on intra-group considerations, including positive feelings about and identification with one’s nation (Li and Brewer, 2004; Greenaway and Cruwys, 2019; Sibley et al., 2020). Studies conducted during the COVID-19 pandemic revealed that the feeling of being part of a group was associated with social cohesion, intention to support others in need, and compliance with prescribed infection-reducing behaviors (Drury et al., 2020; Stevenson et al., 2021). In particular, it has been argued that a stronger social identity is likely to encourage adherence to social norms and fellowship through the mechanism of shared responsibility, which motivates citizens to contribute to collective goals (Haslam et al., 2021). Moreover, Paolini et al. (2020) reported that national identification, identification with Europeans, and identification with humankind – that is, the sense of belonging to the broader category of humans, all of whom are possibly vulnerable to the virus – were positively related to well-being and happiness during the lockdown. Conversely, not having the chance to create a sense of social identity with one’s nation might be related to more negative beliefs. For example, the perception that one’s country was politically divided before the pandemic was found to be associated with negative expectations about the post–COVID-19 vitality of the country and its economy (Crimston and Selvanathan, 2020).

Of course, the coronavirus does not respect borders. Many leaders have observed that effectively reducing its spread or mitigating its negative effects require coordination, cooperation, and solidarity among countries (e.g., European Council, 2020). In this sense, the COVID-19 pandemic has re-ignited the importance of European identity and trust in its institutions to deal with its long-term societal and economic consequences (Caratelli, 2020; Jetten et al., 2020). This is clearly grasped by the words of the European Council President, Charles Michel (European Council, 2020): “This pandemic is putting our societies under serious strain. The well-being of each EU member state depends on the well-being of the whole of the EU. We are all in this together.” It is also worth noticing that European and national identities are not necessarily mutually exclusive: Indeed, they have been found to correlate positively in all European countries except for Britain (Cinnirella, 1997; Cram, 2009). In Italy, as noted by Triandafyllidou (2008), citizens have persistently shown positive responses to survey questions on European identity or feelings of attachment to the EU under both pro-EU and Euro-sceptic governments. Moreover, Italian and European identities appear to be intertwined, formed in a dynamic and interactive relationship (Triandafyllidou, 2008). Some argued that Italians’ identification with their country and with the EU are compatible because they have the most to gain economically and because they perceive the EU to offer a more stable and efficient political structure than the Italian state (e.g., Barzini, 1983; Hewstone, 1986; Cinnirella, 1997). Being either complementary or reinforcing each other, multiple identities allow citizens to simultaneously feel attached to their national state and the EU (Aichholzer et al., 2021). Thus, we argue that social identification, being it with a national or supranational entity such as the EU, might be crucial for how people deal with the coronavirus crisis in the present and also influence their concerns about what will happen in the future.

### The Role of Trust in Institutions Responsible for Managing the Pandemic

A key mechanism through which shared group membership might affect people’s attitudes during the pandemic is trust in the institutions in charge of managing the crisis. In this respect, trust can be intended as the conviction that such institutions are responsive and do what is right for the nation, even in the absence of scrutiny, monitoring, and control by citizens (Miller and Listhaug, 1990). Research showed that individuals who trust politicians and political institutions are more willing to undergo sacrifices for the common good (Im et al., 2014) and comply with government policies (Scholz and Lubell, 1998; Daniele and Geys, 2015). For instance, public trust has been associated with adherence to public health interventions (Meredith et al., 2007) and greater willingness to adopt preventive health behaviors (Rubin et al., 2009; Blair et al., 2017; Verger et al., 2018).

Notably, research on the so-called “rally-round-the-flag” effect (Mueller, 1970) highlighted that, in difficult situations such as terrorist attacks, wars, or pandemics, people set aside political differences and show surging support for the figures or institutions representing the nation under assault (Esaiasson et al., 2020). In line with this contention, several studies revealed increased institutional trust during the COVID-19 crisis in various countries, including Italy (Bol et al., 2020; Falcone et al., 2020; Roccato et al., 2020). In turn, higher level of trust in the government with regard to its ability to control COVID-19 was significantly associated with greater compliance with measures such as frequent hand-washing, avoidance of crowded spaces, and social isolation or quarantine (Han et al., 2021; see also Harring et al., 2021). Similarly, greater trust in science and scientists positively influenced compliance with the COVID-19 prevention guidelines (Plohl and Musil, 2021).

Pagliaro et al. (2021) found that trust in governments, science, and other social actors involved in the crisis – such as citizens– was a better predictor of compliance with prescribed behaviors than information related to the actual threat of the virus (such as the publicized statistics concerning infections and deaths in each country). Moreover, Paolini et al. (2020) reported that Italians’ trust in social and political actors was positively related to well-being during the first phase of the COVID-19 pandemic. The authors also showed that well-being was associated with social identification with meaningful groups, which were modeled as mediators of the effects of trust. However, based on the assumptions of social identity theory, identification with meaningful groups should be considered an antecedent rather than a consequence of trust in institutions (Saleem et al., 2019; Cruwys et al., 2020; for similar reasoning, see Toya and Skidmore, 2014). Indeed, as Brewer (1996) noted, a sort of presumptive trust, not based on personal information, can arise from the awareness of sharing category membership, which leads to attributing positive characteristics to other ingroup members (for similar reasoning, see Kramer, 1999). Interestingly, trust has been proposed to mediate the effect of social identification on cooperative behavior (e.g., Brewer, 1991; La Barbera and Ferrara, 2012). Similarly, Sibley et al. (2020) argued that the enhanced societal trust observed among New Zealanders in the first weeks of the COVID-19 emergency could be due to increased patriotism and the shared need to work together as a society to overcome the crisis. For these reasons, in the present research, we assumed that identification with Italians and Europeans would be positively related to individual levels of positive expectations for the future through enhanced levels of trust in the main institutions having a primary role in managing the crisis.

## The Present Study

This research aimed to shed light on the processes that could explain how people preserved their optimism about the future during the first COVID-19 lockdown. To this end, it examined whether identification with the national group of Italy and with the supranational group of Europe – seen as the key entities and references in the management of the COVID-19 crisis – were associated with more positive expectations about the future in a sample of Italian citizens. In order to capture individuals’ views that the pandemic could have favorable outcomes, positive expectations for the future were declined in a 3-fold way: expectations that Italians, Europeans, and human beings would improve as a consequence of having to cope with the COVID-19 pandemic. Moreover, we tested whether such associations were mediated by trust in the three main institutions involved in managing the pandemic: the Italian government, which directly established anti-covid restrictions and managed the economic and social crisis; the European Union, which was asked to adopt bold economic measures to help the state members and coordinate shared practices (e.g., Caratelli, 2020); and the scientific community, which was entrusted of providing recommendations on how to prevent infections, controlling the spread of the virus, and finding solutions to overcome the pandemic, such as therapies and vaccines (e.g., Fearon et al., 2020; Bicchieri et al., 2021; Pagliaro et al., 2021; Plohl and Musil, 2021).

Data were collected in April 2020, when Italy was struck by a massive death toll (especially in the northern regions of Lombardia, Veneto, and Emilia Romagna) and was on strict lockdown. Based on the premise in social identity theory that the subjective sense of belonging to a collective entity protects against the adverse psychosocial effects of crises (e.g., Haslam et al., 2018; Jetten et al., 2020), we expected that greater social identification with Italians would be positively related to optimistic expectations for the future. It also seems plausible that the feeling of being part of an overarching entity that might be able to give a collective response to the emergency might contribute to boosting people’s morale and optimism regarding the future. Accordingly, we hypothesized that identification with Europeans would be positively associated with positive expectations about the future.

Since greater identification with meaningful groups increases trust in the group and its leaders (Haslam, 2001; Giessner and van Knippenberg, 2008; Cruwys et al., 2020), and trust in institutions is positively related to individuals’ well-being and compliance with rules during crises (Im et al., 2014; Sibley et al., 2020), we also expected that the effects of social identification on respondents’ attitudes toward the future would be mediated by their trust in the Italian government, European Union, and the scientific community.

## Materials and Methods

### Participants

A total of 1,146 Italian participants were recruited through posts on social networking sites and voluntarily took part in the study. Four participants did not provide consent to participate. Another 298 participants were excluded from the analyses because they exited the survey without completing the entirety of the questionnaire, resulting in a final sample of 846 participants (612 women, 234 men; *M*_age_=38.32years, *SD*=14.90years, range 18–79years). Of these, 524 lived in the northern regions of Italy, whereas 322 lived in the central and southern regions; 58 (6.9%) reported that they had contracted the coronavirus, and 191 (22.6%) reported that a family member or a close friend had contracted the virus. For Structural Equation Models (SEM) incorporating latent variables, it is commonly recommended that the ratio of observation to estimated parameters is at least 5:1 (Kelloway, 2015). Given that our model has 70 parameters, the sample size can be considered adequate (see also Soper, 2021).

### Procedure and Measures

This study is based on Wave 1 of a multiple-wave research project for which data collection is still in progress. The project was approved by the Bioethical Committee of the first author’s institution. Wave 1 data were collected in Italy between April 15 and 30, 2020. The questionnaire was administered *via* Qualtrics. Before beginning the questionnaire, participants were provided with relevant information about the study and were informed about their right to anonymity and to stop their participation at any time. After providing their consent to participate, they completed the two identification measures, the trust measures and the two measures related to future expectations. The questionnaire included other measures that are not analyzed in the present paper (e.g., adherence to restriction measures, coping strategies, and well-being). Participants were then asked whether they, a member of their family, or a close friend had contracted the coronavirus. Responses to these two questions were collapsed and indicated that, overall, 224 respondents (26.5%) had a personal experience with COVID-19. Finally, participants were presented with a measure of political orientation and demographic measures.

Identification with Italians and Europeans was measured through two scales adapted from Group Identification Scale of Sani et al. (2015). Each measure comprised three items addressing the respondent’s sense of belonging to the group (e.g., “I have a sense of belonging to Italians/Europeans”; 1=*strongly disagree*, 7=*strongly agree*). Cronbach’s alphas were 0.82 for identification with Italians and 0.91 for identification with Europeans. Participants were then asked to rate how much they trusted that the Italian government, the EU, and the scientific community would be able to manage the coronavirus emergency (1=*not at all*, 5=*a lot*).

Future expectations were measured through five *ad-hoc* items introduced with the instruction: “Thinking of the possible repercussions of the COVID-19 emergency in the next months, please rate the extent to which you disagree or agree with the following items” (1=*completely disagree*, 7=*completely agree*). Two items focused on expectations about people in general (“The pandemic will make better people” and “The pandemic will make the world a better place”; expectations about humankind; *α* =0.80). One item concerned positive expectations about Italian leaders (“The emergency will teach Italian leaders how to cope with new crises”) and two pertained to expectations about the EU (“The emergency will make European Union countries more united” and “The emergency will increase collaboration among European Union nations”; *α*=0.93). Finally, political orientation was measured by asking participants to report their political self-placement on an 11-point left–right continuum (0=*completely left*, 10=*completely right*). The sample mean of political orientation was 3.99 (*SD*=2.20).

## Results

### Preliminary Analyses

Table 1 shows the mean values of all measures in the total sample and as a function of gender, place of residence, and personal experience with COVID-19 infection. Overall, participants showed higher identification with Italians than Europeans, *t*(845)=23.59, *p*<0.001, Cohen’s *d*=0.82. They reported lower trust in the Italian government than in the EU, *t*(845)=−17.61, *p*<0.001, *d*=0.61, or in the scientific community, *t*(845)=−24.77, *p*<0.001, *d*=0.86. Trust in the EU was lower than trust in the scientific community, *t*(845)=−11.78, *p*<0.001, *d*=0.41. Expectations about Italian leaders were more positive than expectations about the EU, *t*(845)=15.68, *p*<0.001, *d*=0.54, or expectations about humankind, *t*(845)=7.77, *p*<0.001, *d*=0.27. Expectations about humankind were more positive than expectations about the EU, *t*(845)=8.72, *p*<0.001, *d*=0.30.

**Table 1:** Descriptive statistics as a function of gender, place of residence, and experience with COVID-19 infection.

	Total	Gender		Place of residence		Experience with COVID-19	
		Women	Men	North	Center-South	Yes	No
Variables	M (SD)	M (SD)	M (SD)	M (SD)	M (SD)	M (SD)	M (SD)
1. Identification with Italians	5.39 (1.05)	5.42 (1.04)	5.32 (1.06)	5.38 (1.02)	5.42 (1.09)	5.34 (1.07)	5.41 (1.04)
2. Identification with Europeans	4.10 (1.55)	4.15 (1.52)	3.96 (1.63)	4.16 (1.53)	4.00 (1.58)	4.05 (1.58)	4.23 (1.46)
3. Trust in the Italian government	2.95 (0.81)	2.96 (0.81)	2.94 (0.82)	2.97 (0.82)	2.93 (0.81)	3.04 (0.84)	2.92 (0.81)
4. Trust in the EU	3.42 (0.83)	3.50 (0.81)	3.21 (0.85)	3.48 (0.80)	3.33 (0.88)	3.50 (3.39)	3.39 (0.84)
5. Trust in the scientific community	3.79 (0.88)	3.78 (0.87)	3.82 (0.90)	3.81 (0.86)	3.75 (0.91)	3.88 (0.82)	3.76 (0.90)
6. Expectations about humankind	3.60 (1.39)	3.66 (1.38)	3.46 (1.42)	3.51 (1.37)	3.75 (1.42)	3.67 (1.40)	3.43 (1.37)
7. Expectations about Italian leaders	3.97 (1.57)	4.06 (1.52)	3.73 (1.67)	3.96 (1.56)	3.99 (1.59)	3.98 (1.57)	3.95 (1.58)
8. Expectations about the EU	3.20 (1.42)	3.24 (1.40)	3.07 (1.45)	3.32 (1.39)	3.15 (1.46)	3.21 (1.42)	3.16 (1.41)

*Measures of identification with Italians, identification with Europeans, and expectations were on a seven-point scale. Trust in institutions was measured on a five-point scale*.

We conducted a series of univariate ANOVAs to assess variations in social identification, trust, or expectations for the future due to participant’s gender, place of residence (northern Italy vs. central and southern Italy), or personal experience with COVID-19 infection. Women reported higher trust in the EU, *F*(1, 844)=21.10, *p*<0.001, *η*^2^=0.024, and more positive expectations about Italian leaders, *F*(1, 844)=7.72, *p*=0.006, *η*^2^=0.009, than men did. Gender had no other significant effects, *F*s<3.71, *p*s>0.055. Participants living in northern Italy showed higher levels of trust in the EU, *F*(1, 844)=6.60, *p*=0.010, *η*^2^=0.008, and lower levels of positive expectations about humankind, *F*(1, 844)=6.11, *p*=0.014, *η*^2^=0.007, than participants living in central and southern Italy. No other differences between northern and southern participants were found, *F*s<1.95, *p*s>0.163. Finally, participants who had personally experienced COVID-19 infection showed slightly higher levels of trust in the Italian government, *F*(1, 844)=3.90, *p*=0.049, *η*^2^=0.005, and more positive expectations about humankind, *F*(1, 844)=4.85, *p*=0.028, *η*^2^=0.006, than participants who had not personally experienced the virus. No other differences emerged, *F*s<3.17, *p*s>0.075.

Table 2 shows the correlations among the study variables, including political orientation and age, which were included as control variables in the mediation model. As can be seen, being politically oriented to the right-wing was negatively related to identification with Europeans, the three trust measures, and expectations about the EU. Age had negative associations with some of the study variables: The older the participants, the lower their levels of identification with Europeans, trust in the three institutions considered, and expectations about Italian leaders.

**Table 2:** Correlations among the study variables.

Measures	1.	2.	3.	4.	5.	6.	7.	8.	9.	10.
1. Identification with Italians	1	0.29^***^	0.28^***^	0.18^***^	0.09^***^	0.24^***^	0.18^***^	0.19^***^	0.04	0.03
2. Identification with Europeans		1	0.36^***^	0.17^***^	0.17^***^	0.15^***^	0.19^***^	0.46^***^	−0.38^***^	−0.10^**^
3. Trust in the Italian government			1	0.56^***^	0.33^***^	0.17^***^	0.34^***^	0.29^***^	−0.23^***^	−0.13^***^
4. Trust in the EU				1	0.43^***^	0.20^***^	0.26^***^	0.22^***^	−0.07^*^	−0.13^***^
5. Trust in the scientific community					1	0.06	0.13^***^	0.12^***^	−0.14^***^	−0.22^***^
6. Expectations about humankind						1	0.58^***^	0.53^***^	0.01	0.06
7. Expectations about Italian leaders							1	0.54^***^	−0.03	−0.12^***^
8. Expectations about the EU								1	−0.16^***^	−0.07^*^
9. Political orientation									1	0.02
10. Age										1

* *p*<0.05;

** *p*<0.01;

*** *p*<0.001.

### Mediation Model

In order to examine the relationships among identification with Italians and Europeans and participants’ expectations regarding the impact of the COVID-19 emergency, we estimated a model in which both identification measures were modeled as predictors and the three measures of expectations were included as parallel outcomes. Trust in the Italian government, the EU, and the scientific community were added as parallel mediators of the two identification measures and the outcome measures. The identification and expectations variables (i.e., the variables composed of two or more items) were latent variables, with items as indicators. The model also estimated correlations between predictors (i.e., social identification), mediators, and outcomes (i.e., expectations for the future). Gender, age, place of residence, experience with COVID-19 infection, and political orientation were included as control variables.

In order to adjust for measurement errors, structural equation modeling with latent variables (Bollen, 1989) was performed using the M-Plus 8.3 program (Muthén and Muthén, 2019). Model parameters were estimated using the maximum likelihood method; mediation was tested by calculating bootstrap estimates (2,000 resamples) of indirect effects together with bootstrapping bias-corrected CIs. The model fit was examined in the overall sample based on various indices (Schumacker and Lomax, 2010). The comparative fit index (CFI) and the Tucker–Lewis index (TLI) should exceed 0.90 to be considered acceptable (Hu and Bentler, 1999). The root mean square error of approximation (RMSEA) and standardized root mean square residual (SRMR) should be less than 0.08 (Hu and Bentler, 1999).

**Figure 1:**
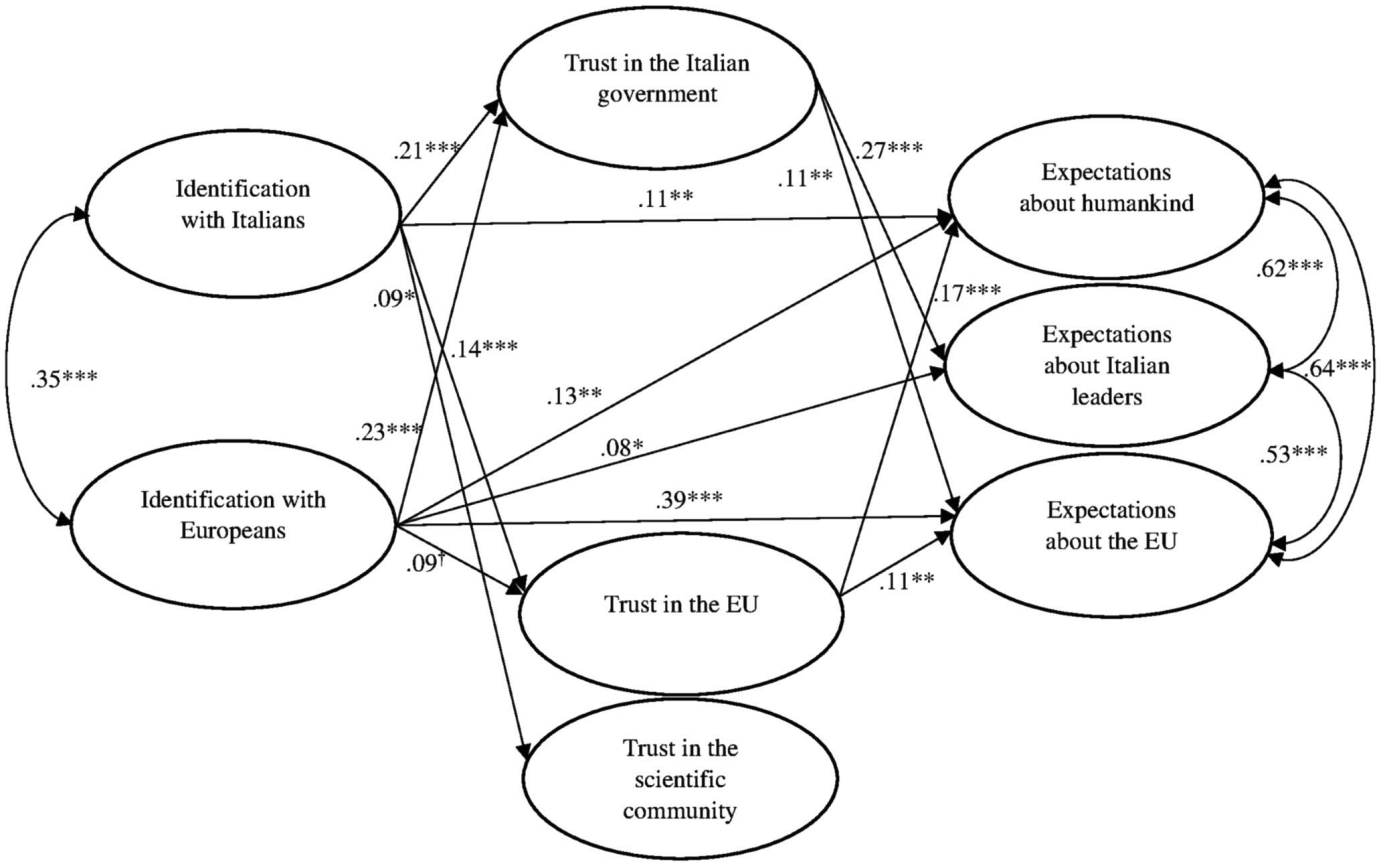
Standardized solution of the model testing the relations among identification with Italians and Europeans, trust in institutions and positive expectations for the future. ^†^*p*=0.053; ^*^p<0.05; ^**^p<0.01; and ^***^p<0.001. Control variables (age, gender, place of residence, political orientation, and experience with COVID-19) were included in the model but are not shown in the figure.

The findings revealed that the model fit was acceptable, CFI=0.955, TLI=0.912, RMSEA=0.068, and SRMR=0.041. The standardized estimates for direct and indirect effects are reported in Table 3. Significant direct links are also shown in Figure 1.

**Table 3:** Standardized Direct and Indirect Effects.

	Trust in the Italian government	Trust in the EU	Trust in the scientific community	Expectations about humankind	Expectations about Italian leaders	Expectations about the EU
	β (SE) [95% CI]	β (SE) [95% CI]	β (SE) [95% CI]	β (SE) [95% CI]	β (SE) [95% CI]	β (SE) [95% CI]
**Direct effects**						
Identification with Italians	0.206^***^ (0.042) [0.123, 0.286]	0.144^**^ (0.047) [0.048, 0.231]	0.087^*^ (0.043) [0.006, 0.170]	0.113^*^ (0.045) [0.025, 0.199]	0.013 (0.040) [−0.064, 0.091]	−0.007 (0.044) [−0.096, 0.077]
Identification with Europeans	0.229^***^ (0.040) [0.148, 0.305]	.085 (0.084) [−0.002, 0.173]	0.082 (0.044) [−0.004, 0.168]	0.132^**^ (0.052) [0.034, 0.236]	0.132^**^ (0.052) [0.000, 0.484]	0.391^***^ (0.046) [0.300, 0.484]
Trust in the Italian government				0.061 (0.052) [−0.038, 0.162]	0.273^***^ (0.044) [−0.007, 0.164]	0.107^*^ (0.046) [0.011, 0.196]
Trust in the EU				0.171^***^ (0.050) [0.069, 0.267]	0.079 (0.044) [−0.007, 0.164]	0.113^**^ (0.043) [0.030, 0.197]
Trust in the scientific community				−0.027 (0.045) [−0.115, 0.060]	−0.016 (0.037) [−0.089, 0.057]	−0.018 (0.039) [−0.093, 0.057]
**Indirect effects**						
Identification with Italians→ Trust in the Italian government→				0.013 (0.011) [−0.008, 0.035]	0.056^***^ (0.014) [0.031, 0.087]	0.022^*^ (0.010) [0.002, 0.043]
Identification with Italians→ Trust in the EU→				0.025^*^ (0.011) [0.006, 0.051]	0.011 (0.008) [−0.001, 0.029]	0.016 (0.009) [0.003, 0.036]
Identification with Italians→ Trust in the scientific community→				−0.002 (0.005) [−0.013, 0.006]	−0.001 (0.004) [−0.010, 0.006]	−0.002 (0.004) [−0.010, 0.005]
Identification with Europeans→ Trust in the Italian government→				0.014 (0.013) [−0.009, 0.040]	0.062^***^ (0.015) [0.036, 0.093]	0.025^*^ (0.012) [0.002, 0.048]
Identification with Europeans→ Trust in the EU→				0.014 (0.009) [0.000, 0.034]	0.007 (0.005) [−0.001, 0.019]	0.010 (0.006) [0.000, 0.023]
Identification with Europeans→ Trust in the scientific community→				−0.002 (0.004) [−0.012, 0.006]	−0.001 (0.004) [−0.009, 0.006]	−0.001 (0.004) [−0.010, 0.006]

* *p*<0.05;

** *p*<0.005;

*** *p*<0.001.

Identification with Italians predicted trust in the Italian government, trust in the EU, and trust in the scientific community. Moreover, identification with Italians had a positive direct association with expectations about humankind, whereas it was not directly related to the other outcome variables. Identification with Europeans was significantly related to trust in the Italian government, whereas its link with trust in the EU was only near significant, and the association with trust in the scientific community was not significant. Identification with Europeans also had direct associations with all the outcome measures.

Considering the indirect effects, identification with Italians had an indirect association with expectations about humankind through trust in the EU. It was also indirectly related to expectations about Italian leaders and expectations about Europe through increased trust in the Italian government.

There were also significant associations among some of the control variables (age, gender, place of residence, and political orientation) and the main measures. Higher age was negatively related to identification with Europe, *β*=−0.064, *SE*=0.032, *p*=0.046, and 95% CI [−0.124, −0.001], trust in the Italian government *β*=−0.104, *SE*=0.033, *p*=0.001, and 95% CI [−0.167, −0.040], trust in the EU, *β*=−0.119, *SE*=0.033, *p*<0.001, and 95% CI [−0.187, −0.051], and trust in the scientific community, *β*=−0.202, *SE*=0.033, *p*<0.001, and 95% CI [−0.267, −0.137]. Age was also negatively related to expectations about Italian leader, *β*=−0.084, *SE*=0.034, *p*=0.013, and 95% CI [−151, −0.019], whereas it was positively related to expectations about humankind, *β*=0.079, *SE*=0.039, *p*=0.045, and 95% CI [0.002, 0.156]. Gender was significantly related to trust in the EU, *β*=0.138, *SE*=0.034, *p*<0.001, and 95% CI [0.071, 0.206], and expectations about Italian leaders, *β*=0.088, *SE*=0.034, *p*=0.010, and 95% CI [0.021, 0.155]. As previously mentioned, women reported higher trust in the EU and higher expectations about Italian leaders than men. Place of residence was significantly related to expectations about humankind, *β*=0.098, *SE*=0.038, *p*=0.011, and 95% CI [0.021, 0.173], which were higher for central and southern respondents. Finally, right-wing respondents were less likely to identify with Europeans, *β*=−0.381, *SE*=0.032, *p*<0.001, and 95% CI [−0.440, −0.317], and showed lower trust in Italian leaders, *β*=−0.148, *SE*=0.036, *p*<0.001, and 95% CI [−0.219, −0.074] and in the scientific community, *β*=−0.112, *SE*=0.038, *p*=0.004, and 95% CI [−0.184, −0.035], compared to left-wing respondents.

## Discussion

The present study examined whether, during the first wave of COVID-19 in Italy, the strength of identification with two meaningful social groups – one’s country and the overarching group of the EU – was related to Italians’ positive expectations about the future. We also analyzed whether these associations were mediated by individuals’ levels of trust in the main institutions in charge of managing the pandemic.

Overall, the findings supported the general assumption that identifications with Italians and Europeans were positively associated with expectations about the future and highlighted the key role of two of the proposed mediators, that is, trust in the Italian government and the EU. Specifically, identification with Italians seemed to feed respondents’ views that the country’s leaders would learn from the management of the pandemic and that the crisis would strengthen the EU by enhancing respondents’ levels of trust in the national government. Moreover, the stronger respondents reported their ties with Italians to be, the more optimistic they were about the possibility that the COVID-19 pandemic would have positive outcomes in terms of the improvement of humankind. This association was partially mediated by increased trust in the EU. Identification with Europeans was directly associated with the three kinds of expectations for the future and was also indirectly associated with expectations about Italian leaders and the EU through increased trust in the Italian government.

Finally, trust in the scientific community was positively correlated with the identification measures as well as with positive expectations about the Italian government and the EU at the bivariate levels, but these relations (expect for the positive association with identification with Italians) were notsignificant when considering the whole model. These findings suggest that trust in the scientific community is likely to rely on other sources. In this respect, it turned out to have interesting negative associations with political orientation – with right-wing oriented respondents showing lower trust than left-wing oriented respondents – and age. Additionally, trust in the scientific community is probably related to other types of outcomes, such as compliance with the COVID-19 containment measures or expectations about the effectiveness of the vaccination campaign (e.g., Bicchieri et al., 2021).

In general terms, the present findings highlighted that both identification with Italians and identification with Europeans contributed to feeding respondents’ optimistic views of the post-pandemic future. At the same time, the results revealed specific patterns through which identifications with the two groups are related to positive expectations. As mentioned, identification with Italians was quite high in absolute terms, and was positively related to expectations about Italian leaders and the EU through increased trust in the Italian government. All in all, the importance of identification with Italians and trust in the Italian government in sustaining expectations for life after the COVID-19 pandemic can be seen as reflecting a rally-round-the-flag effect, that is, the increased support for a country’s government that typically follows the perception of a common threat during international crises (Greenaway and Cruwys, 2019; Moscatelli et al., 2019; Sibley et al., 2020). Moreover, identification with Italians was related to expectations about humankind through increased trust in the EU, whereas the links between identification with Europeans and expectations about Italian leaders and the EU were partially accounted for by trust in the Italian government. Thus, these findings seem to suggest that the two group memberships somehow reinforce respondents’ trust in one another’s institutions, possibly underlining their views and hope that the two groups are interconnected and should reciprocally cooperate in dealing with the crisis. Indeed, the attachments to one’s nation and the superordinate group of Europeans were positively related, likely because, for Italians, membership in the EU fulfills a need for societal modernization and reform of their own country (Balfour and Robustelli, 2019). This finding aligns with previous evidence on the interplay between national and European identification (Cinnirella, 1997; Triandafyllidou, 2008; Kende et al., 2018).

Somehow surprisingly, trust in the EU as an institution – which, in line with pre-pandemic evidence (Serricchio, 2012; Balfour and Robustelli, 2019), was higher than trust in the national government – did not account for the links between identification with Europeans and expectations for the future. We suspect that, at the very beginning of the pandemic, Italians’ traditionally high levels of Europeism and European identification might have been under strain due to the lack of a joint and coordinated reaction by European countries, as well as by the awareness of being the only EU country to be hit severely by the COVID-19 (Falcone et al., 2020; Fontana, 2020). In other words, our findings highlight that the possibility of identifying with and therefore feeling close to the Europeans was the key predictor of expectations about the future. It should also be noted that, consistent with the results of political polls (e.g., Emanuele et al., 2016; Angelucci and Emanuele, 2020), identification with Europeans was related to political orientation, with right-wing respondents less likely to identify with Europe. It was also higher for younger respondents, supporting previous findings of a more positive views of the EU among young people (Wike et al., 2019).

Despite variations due to political orientation and age, the strong direct associations among identification with Europeans and positive for the future underline the importance of the EU membership in facing the pandemic. Not only does the EU have more resources than individual member states in bargaining with drug companies for vaccine supplies and providing financial support to the most hard-hit countries, but it can also help individuals cope with feelings of insecurity and fear related to the pandemic. Namely, individuals’ feelings of belonging to an inclusive group such as the EU can buffer the negative impact of the pandemic at a psychological level.

The results of this study can be interpreted in terms of compensatory strategies intended to restore people’s perceptions of control over events when they have to deal with uncertainty and unpredictability (Pellegrini et al., 2021). In fact, expectations that even the COVID-19 crisis will have positive repercussions might be related to the widely shared assumption that the world is fundamentally just (Lerner, 1980; Tomaka and Blascovich, 1994), implying that good outcomes will somehow compensate for negative events. This reasoning can increase individuals’ willingness to accept tragic events while reducing their need to appeal to other compensatory strategies, such as beliefs in conspiracy theories, to regain control over a threatening reality (Biddlestone et al., 2020; Moscatelli et al., 2021; Pellegrini et al., 2021). Such an account in terms of compensatory strategies does not undermine the novel findings that national and European identification can support individuals’ coping by strengthening positive expectations for the future. Nevertheless, further studies should address this issue and examine whether endorsing positive expectations for the future results in lower feelings of threat.

This study has certain limitations. In particular, relying on cross-sectional data restricts the inferences that can be made concerning the causal direction of the effects. As previously mentioned, based on social identity theory (e.g., Tajfel and Turner, 1986; Jetten et al., 2020), we assumed that social identification functioned as a predictor of trust in the institutions under consideration and, through it, of expectations for the future. Future studies could rule out alternative paths by adopting an experimental design where identifications are experimentally induced or by employing a longitudinal design. These should, however, consider the multiple factors that might intervene in a pandemic crisis, such as fluctuations in contagion due to changes in COVID-19 containment measures, seasonal changes, and vaccination campaigns.

### Theoretical and Practical Implications

From a theoretical perspective, these results add to the literature showing the benefits of social identification for both physical and psychological well-being, coping with crisis, or compliance with health recommendations (e.g., Haslam et al., 2018; Albarello et al., 2019; Jetten et al., 2020). Indeed, the present study highlights that identifying with meaningful social groups might boost people’s resilience in the face of threatening events by sustaining their trust in some of the institutional actors involved in the management of a crisis. Such identifications might help people maintain expectations of a better future, feeding the belief that even a crisis can have some desirable side effects.

These findings have important implications for the management of the coronavirus crisis. First, national leaders should act in ways that boost citizens’ identifications with their national group. At the communicative level, this might be done by stressing that “We are all in this together” and avoiding emphasizing contrasts between subgroups within the same nation (for example, among different political parties). Even though it is very likely that different political parties, different categories of workers, and other specific segments of the population (e.g., older people or the parents of schoolchildren) will have different interests, it is important that governments maintain and reinforce the idea that, despite divergences and the difficulty of making decisions that satisfy all parties, the country is facing a common fate and all involved parties must work toward a common goal. Citizens should feel that they can be proud of their nation and their government without forgetting that they are all “in the same boat” and should pursue a common goal (for similar reasoning, see Scardigno et al., 2021).

Although the EU may be seen as a quite distant entity, as its authorities are often depicted as “rulers” and “bureaucrats” (e.g., Wike et al., 2019; Berti, 2020), it is nevertheless crucial that European leaders put energy into improving communication with EU citizens. Seeing that the EU is cohesive and able to reach collective goals (for instance, when dealing with drug companies to obtain a vaccine supply and supporting member states in need) might reinforce individuals’ sense of belonging to the EU. This was clearly grasped by the President of the European Commission, Ursula von der Leyen, when she called for a common European identity to overcome early setbacks in responding to the pandemic: “When Europe really needed an ‘all for one’ spirit, too many initially gave an ‘only for me’ response” (Wheaton and De La Baume, 2020). As our findings suggest, stronger European identification might be key to tackling fears and challenges related to the pandemic. National leaders should also be aware of the importance of nurturing the sense of being part of a superordinate common group when communicating about EU decisions (Gaertner et al., 2016; Haslam et al., 2021).

In conclusion, this study highlights the crucial role of group identification in fostering individuals’ trust in the institutions in charge of addressing a large-scale crisis like the coronavirus pandemic and in nurturing more positive views of the future. If individuals can count on meaningful shared identities, they will be better equipped to face the prescribed restrictions and even maintain hope that such a crisis will ultimately have some positive outcomes.

## Data Availability Statement

The datasets presented in this study can be found in online repositories. The names of the repository/repositories and accession number(s) can be found at: https://osf.io/td2sr/?view_only=de4c49b6a1f64452ab698bcb8e09a6f3.

## Ethics Statement

The studies involving human participants were reviewed and approved by Bioethical Committee of the University of Bologna. The participants provided their written informed consent to participate in this study.

## Author Contributions

SM, ARG, and LB conceived and designed the study. SM, ARG, LB, SC, and MM collected the data. SM and MM conducted the statistical analyses. All authors wrote the manuscript and contributed to its final version. ARG funded the research.

## Funding

The publication fees were founded by the Fondo di Finanziamento delle Attività Base di Ricerca (FFABR) provided by the Ministero dell’Istruzione, dell’Università e della Ricerca (Legge n. 232 del 11 dicembre 2016, art. 1, cc. 295-302) to ARG.

## Conflict of Interest

The authors declare that the research was conducted in the absence of any commercial or financial relationships that could be construed as a potential conflict of interest.

## Publisher’s Note

All claims expressed in this article are solely those of the authors and do not necessarily represent those of their affiliated organizations, or those of the publisher, the editors and the reviewers. Any product that may be evaluated in this article, or claim that may be made by its manufacturer, is not guaranteed or endorsed by the publisher.
